# Investigation of the Ability of Crocin to Treat Skin Cancer Chemically Induced in Mice via the Inhibition of the Wnt/β-Catenin and Fibrotic Pathway

**DOI:** 10.7759/cureus.38596

**Published:** 2023-05-05

**Authors:** Abdullah Alyoussef

**Affiliations:** 1 Internal Medicine, Faculty of Medicine, University of Tabuk, Tabuk, SAU

**Keywords:** β-catenin, wnt, tumor necrosis factor (tnf)-α, smad, transforming growth factor (tgf)-β, nuclear factor (nf)κb

## Abstract

Background

The Wnt pathway is a major pathway in the pathogenesis of skin cancer. Moreover, crocin is one of the carotenoid compounds present in the flowers of gardenia and crocus. Crocin is responsible for the characteristic color of saffron.

Aims

This study was conducted to discover the therapeutic effects of crocin against skin cancer induced in mice by blocking the Wnt pathway with subsequent effects on inflammation and fibrosis.

Methods

For the induction of skin cancer in mice, the application of DMBA and Croton oil was used. The dorsal skin was used for the evaluation of the gene and protein expression of TGF-β, SMAD, Wnt, β-catenin, TNF-α, and NFκB. Part of the skin is stained with Mallory trichrome.

Results

The use of crocin for treating skin cancer mice significantly reduced both the number of tumors and the number of scratches. In addition, crocin inhibited epidermal hyperplasia. Finally, crocin reduced the gene expression and protein levels of Wnt, β-catenin, SMAD, NFκB; TGF-β and TNF-α.

Conclusions

Crocin produced therapeutic effects against skin cancer induced in mice by blocking the expression of Wnt followed by blocking the pro-inflammatory pathway through downregulation of NFκB and TNF-α. In addition, crocin blocked the fibrosis pathway via the downregulation of TGF-β.

## Introduction

Skin cancer is defined as an irregular enlargement and proliferation of skin cells. It is caused by a cancerous overgrowth of abnormal cells in the basal layer of the epidermis [1]. Skin cancer is a major health problem with increased incidence in all age groups in recent years. Based on the report of the International Agency for Research on Cancer, about 1.518 million new skin cancer cases were associated with 121,000 new deaths worldwide in 2020, leading to a great effect on public health and the economy [2]. Besides surgical excision, many strategies are used in treating skin cancer, including chemotherapy, topical treatments, such as imiquimod and 5-FUlaser, immunotherapy, radiotherapy, cryotherapy, and photodynamic. However, various treatment options exist, but they often have side effects, and drug resistance, and are expensive [3].

Crocin is a carotenoid compound that was obtained from flowers of *Gardenia jasminoides*. It gives saffron its characteristic color. Crocin produces several therapeutic effects such as antipyretic, analgesic, antioxidant, anti-inflammatory, and anticancer [4]. Crocin produced therapeutic effects as well as protective effects against several types of cancer via antioxidant and anti-inflammatory effects (for a more detailed review, see [3]). Searching previous literature revealed that only one study examined the ability of crocin to treat skin cancer. The study reported the ability of crocin to inhibit the proliferation of A431 and SCL-1 skin cells by enhancing apoptosis via blocking the Jak2/Stat3 pathway, repressing the Bcl-2 protein, and increasing the levels of Bid and procaspase-3 [5]. Therefore, this study was conducted to discover the therapeutic effects of crocin against skin cancer chemically induced in mice via blocking the Wnt pathway with subsequent effects on inflammation and fibrosis.

## Materials and methods

Animals and their treatment outlines

For induction of skin cancer, Swiss albino mice aged about seven to eight weeks old and weighing about 26±3 g were used. All mice were preserved in a pathogen-free medium and kept in separate cages with a 12-h light-dark cycle. The mice were kept at a temperature of 22±2°C. All animal procedures were approved by the local ethical committee of the University of Tabuk under number 1438-043. Mice were classified into four groups with 10 mice each:

Control Group

The mice in this group underwent dorsal hair shaving followed by the application of acetone over the shaved area three times weekly over the course of the experiment.

Crocin-Treated Control Group

Mice in the treated control group were treated exactly as those in the control group and were then treated with a subcutaneous injection of 20 mg/kg crocin (Sigma Aldrich Chemicals Co., St. Louise, MO) three times per week for 16 weeks.

Skin Cancer Group

The mice in the skin cancer group underwent dorsal hair shaving. A single dose of 100 mg/100 ml of DMBA (Sigma Aldrich Chemicals Co.) was applied to the shaved area of the back of the mice. Two weeks later, a solution of 1% Croton oil prepared in acetone was used as a promoter for skin cancer by application on the back of the mice three times weekly for 16 weeks.

Crocin-Treated Cancer Group

The mice in this group were treated exactly like the skin cancer group and concurrently treated with a subcutaneous injection of 20 mg/kg crocin three times per week for the whole course of treatment of 16 weeks. The treatment with crocin started exactly from the first week directly with skin cancer induction.

Evaluation of the number of scratches and average number of tumors

In the last two days before mice sacrifice, each mouse was evaluated for the number of scratches in a period of 10 minutes and repeated five times for each mouse. The behavior of scratching was defined as any movement of the hind paws. On the last day before sacrifice, each mouse was investigated carefully to count the average number of tumors or papillomas over the back skin.

Collection of skin samples from mice

At the end of the experiment, the mice were sacrificed by decapitation. The affected parts of the skin, as well as serum samples, were collected and kept at -80°C for further analysis.

Morphologic analysis of skin tissue

The samples of skin collected from mice were then cut into sections with 5-micrometer thickness. The sections were stained with Mallory trichrome stain. The sections were examined in a masked manner.

Enzyme-linked immunosorbent assay (ELISA) determination

The evaluation of components was done by commercially available ELISA. The kits were used for the evaluation of serum values of TNF-α, TGF-β (MyBioSource, San Diego, CA), SMAD3, and β-catenin (Abcam, Cambridge, MA), in accordance with the protocols of the manufacturer. The ELISA plates were read using a microplate reader (BioTek, Winooski, VT).

Quantitative real-time polymerase chain reaction (RT-PCR)

Gene expression of TGF-β, β-catenin, TNF-α, Wnt1, and NFκB were evaluated as described previously [6,7]. Moreover, glyceraldehyde 3-phosphate dehydrogenase (GAPDH) was used as an internal reference. Specific primers are summarized in Table 1.

**Table 1:** The primer sets used

Primer	Accession number	Sequence (sense, antisense)
Wnt1	NM_021279	5`-CGAGAGTGCAAATGGCAATTCCG-3` 5`-GATGAACGCTGTTTCTCGGCAG-3`
β-catenin	NM_001165902	5`-GTTCGCCTTCATTATGGACTGCC-3` 5`-ATAGCACCCTGTTCCCGCAAAG-3`
NFκB	NM_008689	5`- GAAATTCCTGATCCAGACAAAAAC -3` 5`- ATCACTTCAATGGCCTCTGTGTAG -3`
TNF-α	X02611	5`- TACTGAACTTCGGGGTGATTGGTCC -3` 5`- CAGCCTTGTCCCTTGAAGAGAACC -3`
TGF-β	NM_011577	5`-CGGGGCGACCTGGGCACCATCCATGAC-3` 5`-CTGCTCCACCTTGGGCTTGCGACCCAC-3`
GAPDH	M32599	5`- ACCACAGTCCATGCCATCAC -3` 5`- CACCACCCTGTTGCTGTAGCC -3`

Statistical analysis

The expression of mean ± standard error was used as a tool for the expression of descriptive data. Analysis of variance (ANOVA) was used to compare means between groups followed by a post-hoc Bonferroni correction test. Statistical computations were performed by using SPSS version 20 (IBM Corp., Armonk, NY). Statistical significance was predefined as P ≤ 0.05.

## Results

Crocin-attenuated skin cancer-induced increase in the number of tumors and scratches

**Figure 1:**
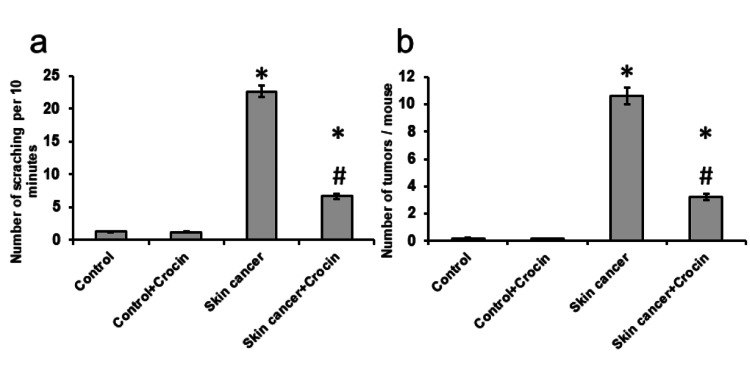
Effect of crocin on skin cancer-induced elevation in the number of scratches per 10 minutes (a) and the number of tumors per mouse (b) *: significant difference as compared with the control groups at p<0.05. #: significant difference as compared with the skin cancer group at p<0.05.

Skin cancer caused a nearly 17-fold elevation in the number of scratches done by each mouse over the period of 10 minutes associated with and a 55-fold increase in the number of tumors found on the back of each mouse, respectively. When the skin cancer mice were treated with crocin, there was about a 70% and 68% reduction in the number of scratches and the number of tumors, respectively (Figure 1).

Effect of crocin on skin cancer morphology

**Figure 2:**
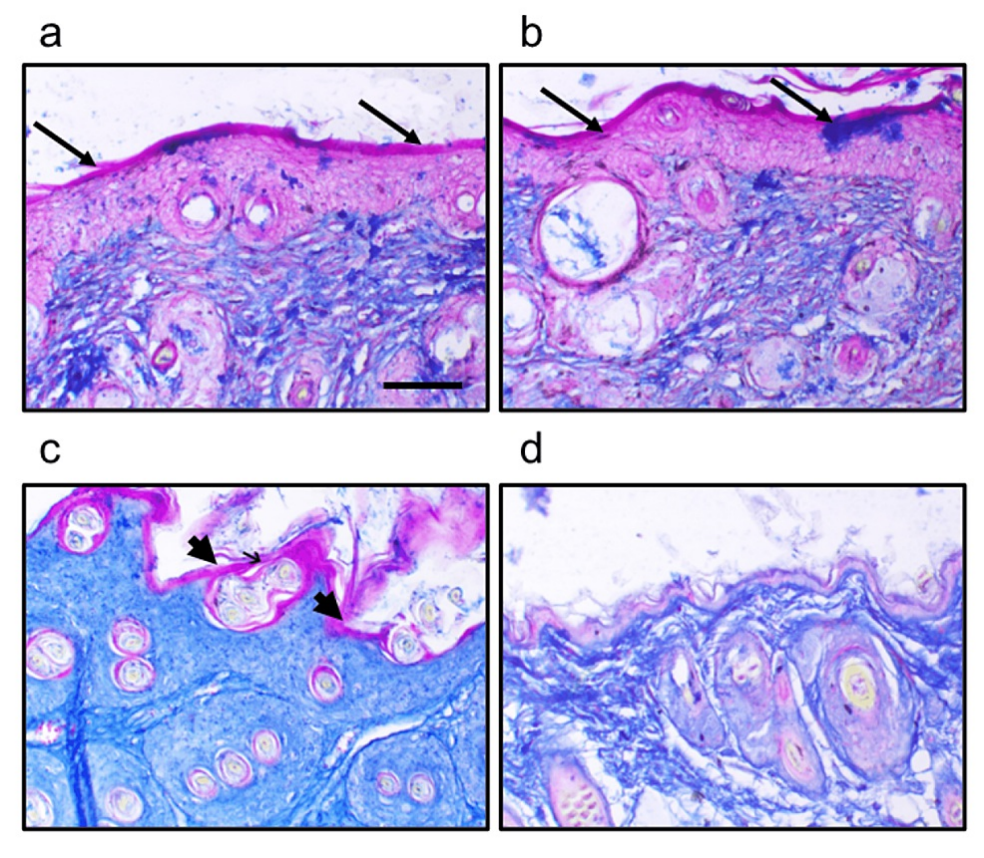
Skin sections stained with Mallory from the control group showing normal epidermis (arrows) (a); control group treated with crocin showing normal epidermis (arrow) (b); skin cancer group showing epidermal hyperplasia, acanthosis, dysplasia, hyperkeratosis (arrowhead), and dermal leukocytic infiltration (arrow) (c); and skin cancer treated with crocin (d) Scale bar 20 μm

The skin sections from mice were stained with Mallory trichrome stain. The control mice sections showed a very narrow layer of keratin and minimal staining in the epidermal layer. On the other hand, an investigation of sections obtained from skin cancer mice revealed hyperkeratosis. The sections from skin cancer mice treated with crocin clearly reduced epithelial hyperkeratosis (Figure 2).

Crocin-blocked skin cancer-induced activation of the Wnt pathway

**Figure 3:**
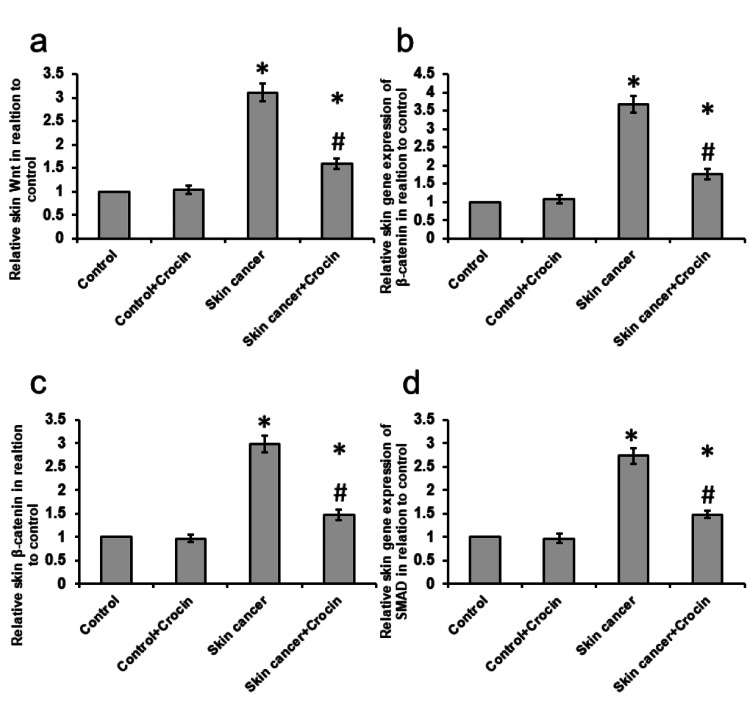
Effect of crocin on the skin cancer-induced gene expression of Wnt (a), β-catenin (b), SMAD (d), and protein level of β-catenin (c) in the skin of mice *: significant difference as compared with the control groups at p<0.05. #: significant difference as compared with the skin cancer group at p<0.05.

Next, we investigated the expression of the Wnt pathway. Skin cancer mice showed about a 3.11-, 3.67- and 2.73-fold elevation in gene expression of Wnt, β-catenin, and SMAD, respectively, connected with a 2.98-fold elevation in protein levels of β-catenin when compared with the control group. Treatment of skin cancer mice with crocin significantly reduced the expression of both Wnt and SMAD in the skin cancer group without affecting the control group (Figure 3).

Crocin-attenuated skin cancer-induced enhancement of inflammation

**Figure 4:**
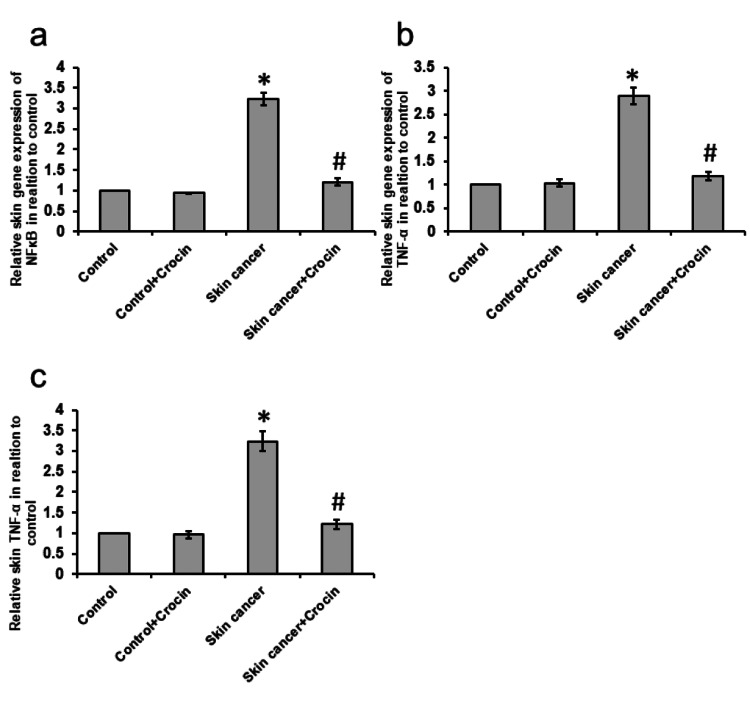
Effect of crocin on skin cancer-induced gene expression of NFκB (a), TNF-α (b), and protein level of TNF-α (c) in the skin of mice *: significant difference as compared with the control groups at p<0.05. #: significant difference as compared with the skin cancer group at p<0.05.

Skin cancer resulted in a 3.23- and 3.67-fold increase in gene expression of NFκB and TNF-α associated with a 2.89-fold elevation in the skin levels of TNF-α as compared with control rats. Treatment of skin cancer mice with crocin significantly reduced the expression of NFκB and TNF-α in the skin samples from skin cancer mice (Figure 4).

Crocin reversed the skin cancer-induced activation of TGF-β

**Figure 5:**
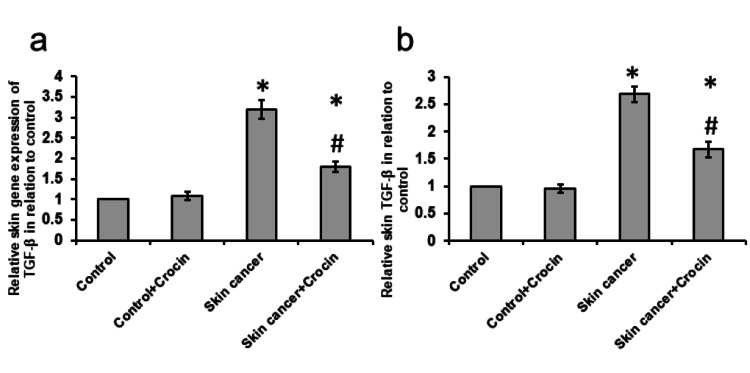
Effect of crocin on skin cancer-induced gene expression of TGF-β (a) and protein expression of TGF-β (b) in the skin of mice *: significant difference as compared with the control groups at p<0.05. #: significant difference as compared with the skin cancer group at p<0.05.

Skin cancer produced a 2.68-fold elevation in gene expression of TGF-β associated with a 3.19-fold increase in the protein levels of TGF-β. In contrast, treatment of skin cancer with crocin significantly reduced the expression of TGF-β (Figure 5).

## Discussion

The current study provided clear evidence of the ability of the natural compound, crocin, to ameliorate skin cancer chemically induced in mice. Crocin significantly reduced the expression of Wnt followed by the deactivation of the pro-inflammatory pathway through the downregulation of NFκB and TNF-α, as well as the attenuation of the fibrotic pathway via the downregulation of the expression of TGF-β. The current manuscript provided promising results in treating skin cancer. During the last decade, skin cancer is considered one of the most aggressive cancers, which is characterized by an elevating prevalence. The disease arises from several risk factors, mainly ultraviolet radiation, family history, and age. It is highly distributed in many countries such as the USA, Australia, and New Zealand. In addition, its prevalence in whites is about 10-fold more than in African Americans [8]. Many therapeutic treatments are available now to deal with the disease, however, they result in minor improvement in the overall survivor rate [9].

There are many molecular mechanisms that are responsible for the initiation, progression, invasion, and metastasis of skin cancer. One of these mechanisms that was illustrated in previous studies is the Wnt/β-catenin pathway that was involved in tumor migration, proliferation, and hematopoiesis. Moreover, the mutation of Wnt/β-catenin was linked to tumor formation and progression [10]. It was also linked to many types of cancer such as skin, prostate, and breast cancers [11]. Therefore, it is a potential therapeutic target in cases of skin cancer. However, β-catenin tried to end gathering inside the nucleus and then it binds to the T-cell factor/lymphoid enhancer binding factor (TCF/LEF) leading to subsequent expression of the target genes and eventually leading to proteasomal degradation [12]. Therefore, it is a predisposing factor in the pathogenicity of skin cancer. In addition, β-catenin activates cell-cell adhesion in cells adjacent to the tumor [10]. We found that treatment of mice with crocin blocked skin cancer-induced overexpression of Wnt and β-catenin connected with amelioration of the symptoms of skin cancer as confirmed by a decrease in tumor number and skin hyperkeratosis, parakeratosis, acanthosis, and dysplasia. Crocin was reported previously to reduce the expression of both Wnt and β-catenin in breast cancer [13] and colorectal cancer [14]. However, no previous study illustrated the ability of crocin to reduce the expression of the Wnt pathway in skin cancer.

Next, we assessed the effect of crocin treatment on the expression of SMADs in skin cancer mice. SMADs are intracellular proteins that help TGF-β signaling and assets in cell signals transfer from receptor to nucleus, followed by the activation of SMAD-binding elements inside the nucleus [15]. They are three major functional classes of SMADs. We measured SMAD3, which belongs to the first class and functions as receptor-regulated SMADS [16]. TGF-β was reported to activate SMAD3 [17].

TGF-β is closely implicated in the progression, invasiveness, and metastasis of skin cancer [18]. TGF-β has two opposite functions in carcinogenesis. In the early stages, it deactivates tumor progression by enhancing the pro-apoptotic genes. However, in later stages, it enhances tumor growth and metastasis [19]. TGF-β inhibits antitumor response by several methods such as inhibiting T-cell differentiation, activating regulatory T cells, and ameliorating tumor-specific cytotoxic T lymphocytes [20]. However, we discovered a reduction in the expression of SMAD3 and TGF-β after treating skin cancer with crocin, which is accompanied by improved skin cell morphology and a reduction in the number of tumors. No previous study illustrated the ability of crocin to reduce the expression of SMAD, however, crocin was reported to reduce the expression of TGF-β in leflunomide-induced liver injury [21], diabetic nephropathy [22], cisplatin (CIS)-induced hepatotoxicity [23], and Bleomycin-induced pulmonary fibrosis [24]. However, no previous study illustrated the ability of crocin to reduce the expression of the TGF-β pathway in skin cancer.

The tumor microenvironment is formed of many types of inflammatory cells such as macrophages, neutrophils, killer cells, mast cells, and T- and B-lymphocytes. They all influence tumor initiation and progression. They also enhance the production of interferons, TNF-α, and interleukins [25]. Moreover, there are many cytokines that could promote tumor development. For example, skin cancer enhances the activity of the NFκB pathway with the subsequent development of drug resistance [26]. NF-κB is considered a leading factor in inflammation, leading to tumorigenesis. In the skin, there are many NFκB-dependent genes that enhance cutaneous inflammation, for example, cytokines, chemokines, and adhesion molecules [27]. One of these factors is TNF-α, which is linked to many procedures of carcinogenesis such as proliferation, angiogenesis, invasion, and metastasis [28]. However, using transgenic mice with genetic deletion of TNF-α is linked to the protection of mice against ultraviolet radiation-induced skin cancer [29]. Finally, we found that treatment of skin cancer mice with crocin blocked the expression of NF-κB and TNF-α.

The limitation of the current study includes that mice have different metabolic pathways and drug metabolites than humans. Another limitation includes the presence of several methods for skin cancer induction in mice. However, only one method is used for the induction of skin cancer by using DMBA. In addition, we used the ELISA and PCR methods for the assessment of the expression of inflammatory and fibrotic pathways, which has some limitations such as sequence accuracy, amplification yield, low signal intensity, and high background.

**Figure 6:**
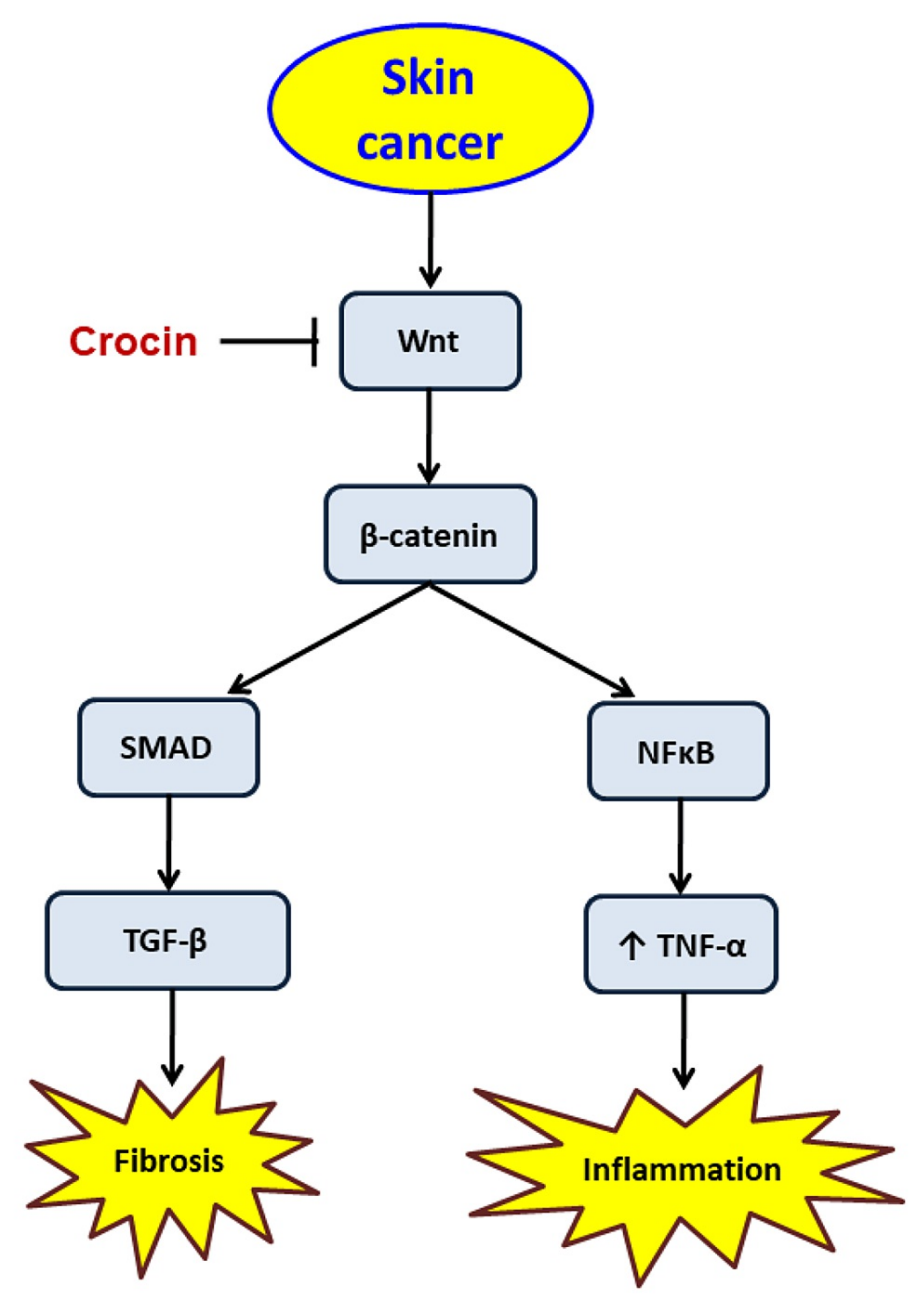
The mechanism of the protective effects of crocin in skin cancer NFκB, nuclear factor κB; TGF-β, transforming growth factor-β; TNF-α, tumor necrosis factor-α.

The therapeutic effects of crocin on skin cancer open a new field for new safe products, highly available in nature and cheap. More studies are needed to make sure of the mechanism of action. Finally, the mechanism of action of crocin in skin cancer is summarized in Figure 6.

## Conclusions

Crocin produced therapeutic effects against skin cancer induced in mice by blocking the expression of Wnt. The blocking of Wnt resulted in the deactivation of the pro-inflammatory pathway through the downregulation of NFκB and TNF-α. In addition, crocin blocked the fibrotic pathway via the downregulation of both the gene and protein expression of TGF-β. The current manuscript provided promising results in treating skin cancer.

## References

[1] Wako BD, Dese K, Ulfata RE, Nigatu TA, Turunbedu SK, Kwa T (2022). Squamous cell carcinoma of skin cancer margin classification from digital histopathology images using deep learning. Cancer Control.

[2] Liu L, Qi M, Li Y, Liu Y, Liu X, Zhang Z, Qu J (2022). Staging of skin cancer based on hyperspectral microscopic imaging and machine learning. Biosensors (Basel).

[3] Bastani S, Vahedian V, Rashidi M (2022). An evaluation on potential anti-oxidant and anti-inflammatory effects of crocin. Biomed Pharmacother.

[4] Sung YY, Lee AY, Kim HK (2014). The Gardenia jasminoides extract and its constituent, geniposide, elicit anti-allergic effects on atopic dermatitis by inhibiting histamine in vitro and in vivo. J Ethnopharmacol.

[5] Wang G, Zhang B, Wang Y, Han S, Wang C (2018). Crocin promotes apoptosis of human skin cancer cells by inhibiting the JAK/STAT pathway. Exp Ther Med.

[6] Alyoussef A, Taha M (2019). Blocking Wnt as a therapeutic target in mice model of skin cancer. Arch Dermatol Res.

[7] El-Far YM, Khodir AE, Emarah ZA, Ebrahim MA, Al-Gayyar MM (2022). Chemopreventive and hepatoprotective effects of genistein via inhibition of oxidative stress and the versican/PDGF/PKC signaling pathway in experimentally induced hepatocellular carcinoma in rats by thioacetamide. Redox Rep.

[8] Rogers HW, Weinstock MA, Harris AR, Hinckley MR, Feldman SR, Fleischer AB, Coldiron BM (2010). Incidence estimate of nonmelanoma skin cancer in the United States, 2006. Arch Dermatol.

[9] Berrocal A, Cabañas L, Espinosa E (2014). Melanoma: diagnosis, staging, and treatment. Consensus group recommendations. Adv Ther.

[10] Li J, Fang R, Wang J, Deng L (2018). NOP14 inhibits melanoma proliferation and metastasis by regulating Wnt/β-catenin signaling pathway. Braz J Med Biol Res.

[11] Gao D, Chen HQ (2018). Specific knockdown of HOXB7 inhibits cutaneous squamous cell carcinoma cell migration and invasion while inducing apoptosis via the Wnt/β-catenin signaling pathway. Am J Physiol Cell Physiol.

[12] Benoit YD, Guezguez B, Boyd AL, Bhatia M (2014). Molecular pathways: epigenetic modulation of Wnt-glycogen synthase kinase-3 signaling to target human cancer stem cells. Clin Cancer Res.

[13] Arzi L, Farahi A, Jafarzadeh N, Riazi G, Sadeghizadeh M, Hoshyar R (2018). Inhibitory effect of crocin on metastasis of triple-negative breast cancer by interfering with Wnt/β-catenin pathway in murine model. DNA Cell Biol.

[14] Amerizadeh F, Rezaei N, Rahmani F (2018). Crocin synergistically enhances the antiproliferative activity of 5-flurouracil through Wnt/PI3K pathway in a mouse model of colitis-associated colorectal cancer. J Cell Biochem.

[15] Massagué J (2012). TGFβ signalling in context. Nat Rev Mol Cell Biol.

[16] Massagué J (1998). TGF-beta signal transduction. Annu Rev Biochem.

[17] Dantonio PM, Klein MO, Freire MR, Araujo CN, Chiacetti AC, Correa RG (2018). Exploring major signaling cascades in melanomagenesis: a rationale route for targetted skin cancer therapy. Biosci Rep.

[18] Mohammad KS, Javelaud D, Fournier PG (2011). TGF-beta-RI kinase inhibitor SD-208 reduces the development and progression of melanoma bone metastases. Cancer Res.

[19] Berking C, Takemoto R, Schaider H, Showe L, Satyamoorthy K, Robbins P, Herlyn M (2001). Transforming growth factor-beta1 increases survival of human melanoma through stroma remodeling. Cancer Res.

[20] Yang L, Pang Y, Moses HL (2010). TGF-beta and immune cells: an important regulatory axis in the tumor microenvironment and progression. Trends Immunol.

[21] Sokar SS, Alkabbani MA, Akool ES, Abu-Risha SE (2022). Hepatoprotective effects of carvedilol and crocin against leflunomide-induced liver injury. Int Immunopharmacol.

[22] Jaafarinia A, Kafami B, Sahebnasagh A, Saghafi F (2022). Evaluation of therapeutic effects of crocin in attenuating the progression of diabetic nephropathy: a preliminary randomized triple-blind placebo-controlled trial. BMC Complement Med Ther.

[23] Khedr LH, Rahmo RM, Farag DB, Schaalan MF, El Magdoub HM (2020). Crocin attenuates cisplatin-induced hepatotoxicity via TLR4/NF-κBp50 signaling and BAMBI modulation of TGF-β activity: Involvement of miRNA-9 and miRNA-29. Food Chem Toxicol.

[24] Zaghloul MS, Said E, Suddek GM, Salem HA (2019). Crocin attenuates lung inflammation and pulmonary vascular dysfunction in a rat model of bleomycin-induced pulmonary fibrosis. Life Sci.

[25] Ferris RL (2015). Immunology and immunotherapy of head and neck cancer. J Clin Oncol.

[26] Ghosh K, Capell BC (2016). The senescence-associated secretory phenotype: critical effector in skin cancer and aging. J Invest Dermatol.

[27] Bell S, Degitz K, Quirling M, Jilg N, Page S, Brand K (2003). Involvement of NF-kappaB signalling in skin physiology and disease. Cell Signal.

[28] Sánchez-Zauco N, Torres J, Gómez A (2017). Circulating blood levels of IL-6, IFN-γ, and IL-10 as potential diagnostic biomarkers in gastric cancer: a controlled study. BMC Cancer.

[29] Singh A, Singh A, Bauer SJ, Wheeler DL, Havighurst TC, Kim K, Verma AK (2016). Genetic deletion of TNFα inhibits ultraviolet radiation-induced development of cutaneous squamous cell carcinomas in PKCε transgenic mice via inhibition of cell survival signals. Carcinogenesis.

